# 新局面下对血液肿瘤药物创新研发的思考

**DOI:** 10.3760/cma.j.issn.0253-2727.2021.12.001

**Published:** 2021-12

**Authors:** 凌 唐, 小明 陈, 丽敏 邹, 瑜 杜, 玥丽 齐, 志敏 杨

**Affiliations:** 国家药品监督管理局药品审评中心，北京 100022 Center for Drug Evaluation, National Medical Products Administration, Beijing 100022, China

自2015年国务院发布《关于改革药品医疗器械审评审批制度的意见》以来，国家药品监督管理局（National Medical Products Administration, NMPA）的药物审评审批不断提速，大大促进了我国新药，特别是抗肿瘤药物的研发。在血液肿瘤领域，新药研发使中国的血液肿瘤患者更早地接受更新、更好的治疗。与国际上新药的同步研发也使国内外临床用药的差异逐步缩小。血液肿瘤药物研发提速的新局面下，对于药物创新研发也需有新的思路、新的思考。

一、血液肿瘤领域药物研发现状

近年来血液肿瘤领域药物的临床试验（Investigational New Drug, IND）申请呈现平稳增长趋势。按受理号计算，2018年共受理血液肿瘤领域药物的IND申请为47项，2020年达到174项，而2021年截至7月30日，已达到165项，接近2020年全年水平。

从全球范围内，截至2021年7月，以“lymphoma”（淋巴瘤）为例作为关键词在www.clinical.org网站检索，全球共登记研究为7457项，中国大陆登记为944项，占12.7%。

临床试验如火如荼，最终的目标是转化为药品上市，以满足患者的治疗需求。近年来，我国血液肿瘤领域获批上市药物呈递增趋势，2018年和2019年血液肿瘤领域获批的化学药物/生物制品上市申请（New Drug Application, NDA）共5个，而2020年一年获批的NDA就达到12个。自2020年1月至2021年7月，除嵌合抗原受体T细胞（CAR-T细胞）治疗以外，NMPA共批准了19项用于治疗血液肿瘤的NDA（表1），美国食品药品监督管理局（FDA）共批准22项（表2）。

虽然FDA所批准的药物仍有部分未在中国上市，但其中大多数产品在中国申报了临床试验，或已申报/计划申报上市，并且这些药物所涉及的靶点/作用机制，在我国均有同靶点/同机制药物在开展临床试验。

**表1:** 国家药品监督管理局2020年1月至2021年7月批准的血液肿瘤药物（不包括CAR-T细胞治疗）

序号	药品名称	适应证
1	阿伐曲泊帕片	用于择期行诊断性操作或者手术的成年慢性肝病患者相关的血小板减少症
2	注射用维布妥昔单抗	治疗复发或难治性经典型霍奇金淋巴瘤患者；治疗复发或难治性系统性间变性大细胞淋巴瘤患者
3	泽布替尼胶囊	适用于既往至少接受过一种治疗的套细胞淋巴瘤患者的治疗
4	泽布替尼胶囊	既往至少接受过一种治疗的慢性淋巴细胞白血病/小淋巴细胞淋巴瘤患者的治疗
5	普拉曲沙注射液	用于治疗复发性或难治性外周T细胞淋巴瘤
6	普乐沙福注射液	与G-CSF联用，适用于非霍奇金淋巴瘤和多发性骨髓瘤患者动员造血干细胞（HSC）进入外周血，以便于完成HSC采集与自体移植
7	利妥昔单抗注射液	生物类似药
8	来那度胺胶囊	与利妥昔单抗合用治疗复发或难治性惰性淋巴瘤
9	维奈克拉片	与阿扎胞苷或地西他滨或低剂量阿糖胞苷联用适于治疗年龄75岁及以上或因合并症不适于接受强诱导化疗的新诊断的成人急性髓系白血病
10	注射用倍林妥莫双抗	用于治疗复发或难治性前体B细胞急性淋巴细胞白血病
11	奥布替尼片	适用于既往至少接受过一种治疗的套细胞淋巴瘤患者的治疗
12	奥布替尼片	既往至少接受过一种治疗的慢性淋巴细胞白血病/小淋巴细胞淋巴瘤患者的治疗
13	富马酸吉瑞替尼片	用于治疗携带FLT3突变的复发性或难治性急性髓系白血病成人患者
14	注射用维布妥昔单抗	为靶向CD30的抗体偶联药物，适用于治疗以下CD30阳性淋巴瘤成人患者：复发或难治性系统性间变性大细胞淋巴瘤；复发或难治性经典型霍奇金淋巴瘤。既往经治的皮肤T细胞淋巴瘤患者
15	达雷妥尤单抗注射液	与来那度胺和地塞米松联合用药或与硼替佐米和地塞米松联合用药治疗既往至少接受过一线治疗的多发性骨髓瘤成人患者
16	奥妥珠单抗注射液	与化疗联用，随后用于维持治疗，用于初治的滤泡性淋巴瘤患者
17	海曲泊帕乙醇胺片	用于对免疫抑制治疗疗效不佳的重型再生障碍性贫血患者的治疗
18	海曲泊帕乙醇胺片	适用于成人原发免疫性血小板减少症患者
19	泽布替尼胶囊	适用于治疗成人华氏巨球蛋白血症患者

**表2:** 美国食品药品监督管理局2020年1月至2021年7月批准的血液肿瘤药物（不包括CAR-T细胞治疗）

序号	产品	适应证
1	loncastuximab tesirine-lpyl	弥漫大B细胞淋巴瘤
2	isatuximab-irfc	多发性骨髓瘤
3	axicabtagene ciloleucel	难治或复发的滤泡性淋巴瘤
4	melphalan flufenamide	难治或复发的多发性骨髓瘤
5	umbralisib	边缘区淋巴瘤和滤泡性淋巴瘤
6	Darzalex Faspro	新诊断的轻链淀粉样变
7	crizotinib	儿童及青少年难治复发的系统性间变大细胞淋巴瘤
8	selinexor	难治或复发的多发性骨髓瘤
9	venetoclax	急性髓系白血病
10	pembrolizumab	经典型霍奇金淋巴瘤
11	Onureg (azacitidine tablets)	急性髓系白血病
12	carfilzomib and daratumumab with dexamethasone	多发性骨髓瘤
13	belantamab mafodotin-blmf	多发性骨髓瘤
14	tafasitamab-cxix	弥漫大B细胞淋巴瘤
15	decitabine and cedazuridine	骨髓增生异常综合征
16	selinexor	难治复发弥漫大B细胞淋巴瘤
17	tazemetostat	滤泡性淋巴瘤
18	gemtuzumab ozogamicin	CD33阳性的儿童急性髓系白血病
19	daratumumab,hyaluronidase-fihj	多发性骨髓瘤
20	ibrutinib plus rituximab	慢性淋巴细胞白血病
21	luspatercept-aamt	骨髓增生异常综合征所致贫血
22	isatuximab-irfc	多发性骨髓瘤

二、快速发展中的不足

快速发展的同时，我国血液肿瘤领域药物研发的“短板”也逐步显现。

1. 创新力有待提高：虽然国内外的药物研发差距在逐步缩小，但我国国产药物仍以fast-follow、me too/me better为主，这也在一定程度上导致了研发“扎堆”，例如程序性死亡受体-1（programmed cell death-1, PD-1）单克隆抗体，目前在我国已有5个PD-1单克隆抗体获批或即将获批难治复发经典型霍奇金淋巴瘤（cHL）适应证。再如布鲁顿酪氨酸激酶（Bruton Tyrosine Kinase, BTK）抑制剂，我国已有两款国产BTK抑制剂上市，已分别附条件获得套细胞淋巴瘤、慢性淋巴细胞白血病/小淋巴细胞淋巴瘤、华氏巨球蛋白血症等适应证，而同时仍有多个BTK抑制剂在递交首次人体临床试验申请（first in human，FIH），并仍以淋巴瘤作为目标适应证进行开发，目前在我国已有约10款BTK抑制剂（不包括已上市的两款BTK抑制剂）在研。

2. 单臂研究“扎堆”：2014至2020年期间，在肿瘤治疗领域，我国共有20项NDA是基于单臂研究获得附条件批准上市，其中9项均为淋巴瘤领域相关适应证，仅“复发或难治性经典型霍奇金淋巴瘤”一个适应证的附条件申请就有3项。血液肿瘤相较于实体瘤，往往客观缓解率（objective response rate，ORR）更高，以PD-1单抗治疗难治/复发cHL为例，在某些产品的关键单臂研究中，ORR可接近90%。较高的ORR使得血液肿瘤领域单臂研究的成功率更高，研发单位也更倾向于采用单臂试验支持抗血液肿瘤药物加速上市。然而单臂试验本身存在较多不确定性，近期在美国多个药物因确证性对照研究未能成功，而撤销了其以单臂试验加速批准的相关适应证。例如罗米地辛在2011年基于两项临床试验中总缓解率替代终点结果，获得美国FDA的加速批准，作为单药治疗既往接受过至少一种系统治疗的外周T细胞淋巴瘤（PTCL）成年患者。随后，百时美施贵宝公司开展了一项验证性Ⅲ期临床试验，评估罗米地辛+CHOP（Ro-CHOP）与 CHOP（环磷酰胺、多柔比星、长春新碱、泼尼松）在一线治疗PTCL患者中的疗效，然而该研究未达到无进展生存期的主要疗效终点，百时美施贵宝公司于2021年8月宣布撤回该适应证。

三、新局面下的研发思考

在血液肿瘤领域，我国与国外临床用药差异在不断缩小，并逐步实现向同步研发过渡；新的研发局面逐步形成，为我国血液肿瘤领域的药物研发带来机遇的同时，也带来了新挑战。

1. 解放思想，推动创新：目前我国药物研发的创新力仍有不足，如何促进我国自主创新是值得思考的重要问题。以往谈到“创新”似乎默认等同于“first in class”。目前过分强调我国自主研发“first in class”药物仍有一定难度，这也是形成当下“me too”、“fast follow”模式为主的原因之一。然而，“创新”绝不是“首个”，更不是“唯一”。2021年7月2日，NMPA药品审评中心发布《以临床价值为导向的抗肿瘤药物临床研发指导原则（征求意见稿）》[1]，进一步强调了“以临床价值为导向”的研发思路。以患者临床需求为核心，以临床价值为导向的各类研发，都可以是“创新”的方向。

患者的需求是“创新”的驱动力。药物研发的起点，一定是患者的治疗需求，研发的持续创新，则源于患者需求的动态变化。BTK抑制剂的研发过程，就较好地阐释了这样的过程。

在BTK抑制剂上市之前，B细胞来源的恶性淋巴瘤一般以非靶向的化疗药物治疗为主。基础研究发现，BTK是调节B细胞增殖和存活的抗原依赖性B细胞受体（B cell receptor，BCR）信号的重要组成部分，这为BTK抑制剂研发提供了理论基础。而由于三磷酸腺苷（ATP）的活性位点高度保守，这导致开发选择性、竞争性ATP酶抑制剂非常困难，进一步研究发现亲电性抑制剂可克服上述难题，因此通过对多个分子结构的筛选，最终开发了亲电基团处于更佳位置，可与位于ATP结合位点近端的Cys481共价不可逆相结合的首个BTK抑制剂伊布替尼[2]。伊布替尼于2013年首次获批上市，而它的上市为B细胞恶性肿瘤迈入无化疗的精准治疗时代创造了可能。

伊布替尼的上市是以“first in class”进行的创新突破，然而出血、心房扑动等不良反应，增加了用药的安全性风险，而后续我国自主研发了泽布替尼、奥布替尼，其安全性特征与伊布替尼不同，在临床试验中观察到的心房扑动/心房颤动、出血的不良反应发生率更低，它们的上市为淋巴瘤患者提供了更多的治疗选择。因此，抗肿瘤药物的创新，不只有机制创新或疗效提高，满足不同患者对药物安全性的不同需求，也是重要的创新。

从伊布替尼到泽布替尼、奥布替尼，对于B细胞恶性淋巴瘤患者而言，实现了“无药可治”到“有药可治”，进而到“有药可选”。在此之后，随着BTK抑制剂的广泛应用，耐药问题随之而来。耐药是抗肿瘤治疗失败的主要原因之一；一旦发生耐药，患者可能又将回到“无药可治”的状态。现有上市BTK抑制剂的结合位点的C481S突变是目前已知BTK抑制剂耐药机制之一；针对该耐药问题，Pirtobrutinib、Fenebrutinib、MK-1026等多个针对C481S突变的BTK抑制剂已进入临床研究阶段；这些新药的研发，将有望满足随着治疗而新产生的、耐药患者的治疗需求。

2. 聚焦精准治疗：精准治疗是以患者为核心新药研发的重要内容。通过精准治疗，可以实现为最合适的患者提供最恰当的治疗。检测技术的发展推动了对肿瘤的认识，从细胞、组织学，到分子、免疫表型，再到驱动基因的层层深入，构建了精准治疗的基础。以弥漫大B细胞淋巴瘤（DLBCL）为例，最初根据细胞起源将其分为生发中心B细胞样（GCB）亚型和非生发中心B细胞样（non-GCB）亚型，而现在已在尝试根据DLBCL遗传学特征的新的基因分类开展研究[3]，亦有药物在基于新的基因分型开展精准人群的临床试验，并有望以此叩开DLBCL精准治疗的大门。

在肿瘤治疗领域，生物标志物是实现精准治疗的重要工具之一。生物标志物通常是指能被客观测量和评价，反映生理或病理过程，以及对暴露或治疗干预措施产生生物学效应的指标。2021年NMPA药品审评中心也发布了《生物标志物在抗肿瘤药物临床研发中应用的技术指导原则（征求意见稿）》[4]。在血液肿瘤领域也有基于生物标志物筛选患者人群的药物获批上市。

研发经验表明，通过有效的生物标志物精准筛选潜在获益人群，有助于提高临床试验成功率，同时还能更加精准满足特定人群的治疗需求。

急性髓系白血病（AML）的传统疾病分型，是根据骨髓细胞形态学特征，将其分为M_0_至M_7_共8个亚型，除了急性早幼粒细胞白血病（M_3_）亚型以外，其余亚型的治疗主要以化疗为主，临床中主要根据患者的基础状态决定相应的化疗方案。然而，研究发现，大约有三分之一的AML患者存在FLT3基因突变，相对于FLT3突变阴性患者，FLT3突变阳性难治复发 AML患者接受挽救性化疗后缓解率更低，至第二次复发的缓解持续时间更短，且总生存期更短[5]。

上述基础研究提示我们，现有治疗对于伴有FLT3突变的AML患者疗效相对差，因此这部分患者对于更有效的治疗有更高的需求。针对这种临床需求，相继有多个FLT3抑制剂进入研发。米哚妥林是首个用于治疗伴有FLT3突变的AML的FLT3抑制剂，在早期研究中就显示出其在伴有FLT3突变的AML中疗效更有优势[6]。2017年4月美国FDA批准米哚妥林用于与化疗联合治疗伴有FLT3突变的AML初治患者。在米哚妥林之后，另一款FLT3抑制剂富马酸吉瑞替尼（Gilteritinib）于2018年获得美国FDA批准上市，获批单药用于治疗伴有FLT3突变的难治复发AML，该适应证于2021年也在我国获得批准。

3. 持续开发，维持药物生命周期活力：创新不是追求“从无到有”。现阶段，充分挖掘已上市产品以往未被充分认识的机制、开发新适应证，都是顺应患者治疗需求的、可行的创新途径。

对产品的持续开发，“老药新用”是创新研发的模式之一。例如沙利度胺，最初作为孕妇妊娠止吐药物上市，而后大约有10 000名儿童出现先天性畸形，造成了药物研发史中最大的悲剧。1961年沙利度胺撤出了市场。然而，后续的研究发现，沙利度胺具有免疫调节作用，能够治疗麻风结节性红斑，由此再次开启了对沙利度胺的开发，1998年7月美国FDA批准沙利度胺上市用于治疗麻风结节性红斑的皮肤表现。此外，D'Amato等[7]在兔体内观察到沙利度胺可以抑制由FGF2介导的血管新生，而血管新生是骨髓瘤患者骨髓瘤细胞增殖必不可少的因素，沙利度胺因此被开发用于治疗复发性多发性骨髓瘤（MM），并表现出了较高的活性。2006年FDA批准沙利度胺用于MM的治疗[8]。

再如西达本胺，该药物是我国拥有自主知识产权的首个组蛋白去乙酰化酶（HDAC）抑制剂。2016年西达本胺基于一项单臂研究获批用于既往至少接受过一次全身化疗的复发或难治的PTCL患者。在对其作用机制的进一步探索发现，西达本胺不仅可以直接诱导肿瘤细胞周期阻滞和凋亡，并且可逆转上皮细胞-间充质转化，抑制肿瘤的转移，还具有乳腺癌特异性抗肿瘤机制，对于HR阳性乳腺癌，西达本胺能抑制旁路生长因子受体信号通路的代偿性活化，逆转内分泌耐药，并且可以通过提高ER某些位点的乙酰化水平，从而直接抑制配体依赖性活化通路。在机制研究的基础上，后续开展的临床研究进一步证实了西达本胺联合芳香化酶抑制剂使乳腺癌患者获益。2019年11月29日，NMPA正式批准西达本胺联合芳香化酶抑制剂用于激素受体阳性、人表皮生长因子受体-2阴性、绝经后、经内分泌治疗复发或进展的局部晚期或转移性乳腺癌患者。

对剂型进行改良，也是“老药”创新的重要方式。例如硼替佐米在原有静脉注射制剂的基础上，开发皮下注射制剂，在有效性相似的情况下，皮下注射给药后2级或2级以上周围神经病的发生率明显降低（静脉注射8%，皮下注射2%，*P*＝0.001）[9]；再如达雷妥尤单抗由静脉注射剂改为皮下注射剂后，输液反应的发生率由28%～50%降至16%；随着输注时间的明显缩短，接受达雷妥尤单抗皮下制剂治疗的患者，对治疗的满意度也高于达雷妥尤单抗静脉制剂组[10]。除了改变给药途径以外，白蛋白制剂、脂质体制剂、纳米技术等也被广泛应用，通过制剂改造，可以改变药物在体内的PK及组织分布行为，提高治疗指数，从而实现提高疗效或改善安全性的目的。上述制剂剂型的改良，不仅改善了患者的治疗便利性，提高依从性，同时还改善了治疗的安全性。

4. 临床试验的创新：血液肿瘤往往缓解率较高，因此很多企业都希望通过单臂研究支持产品上市。事实上，采用单臂研究并不一定是最高效的研发策略；高效研发的思路应贯穿整个研发过程，而非仅仅集中在支持上市的关键研究设计。

在研发过程中，积极探索替代终点，可助力加速研发。例如微小残留病（MRD）作为替代终点的潜力正被广泛关注。对于血液肿瘤而言，病理取材较实体瘤而言相对简便，因此也更具备开发MRD的潜质，FDA于2020年1月发布血液恶性肿瘤中应用MRD的相关指导原则[11]，NMPA药品审评中心自2020年以来，也发布了一系列相关指导原则或指导原则征求意见稿。

研发中应充分利用科学工具。例如应用模型引导的药物开发（model-informed drug development，MIDD），可最大可能地降低肿瘤患者接受无效剂量、非充足剂量治疗的可能，同时用尽可能少的肿瘤受试者完成研究目的，也将大大提高研发效率[12]。

四、总结

在血液肿瘤领域，新药研发提速。快速发展的同时，我国新药研发创新力仍有不足。机制创新是发展的根本动力，加强基础研究的同时，我们更应解放思想，把药物创新从单纯追求“first in class”的固有思维中跳出来。满足患者治疗需求是药物研发的终极目标，因此以患者需求为核心，以临床价值为导向，从多维度进行思考，将会发现更多的创新点。
